# Genome-Wide Identification of the *SUN* Gene Family in Melon (*Cucumis melo*) and Functional Characterization of Two *CmSUN* Genes in Regulating Fruit Shape Variation

**DOI:** 10.3390/ijms232416047

**Published:** 2022-12-16

**Authors:** Ming Ma, Suya Liu, Zhiwei Wang, Ran Shao, Jianrong Ye, Wei Yan, Hailing Lv, Agula Hasi, Gen Che

**Affiliations:** Key Laboratory of Herbage & Endemic Crop Biology, Ministry of Education, School of Life Sciences, Inner Mongolia University, Hohhot 010070, China

**Keywords:** melon, CmSUN, IQ67 domain, expression analysis, overexpression phenotype, fruit shape regulation, protein interaction

## Abstract

Melon (*Cucumis melo*) is an important economic crop cultivated worldwide. A unique *SUN* gene family plays a crucial role in regulating plant growth and fruit development, but many *SUN* family genes and their function have not been well-characterized in melon. In the present study, we performed genome-wide identification and bioinformatics analysis and identified 24 *CmSUN* family genes that contain integrated and conserved IQ67 domain in the melon genome. Transcriptome data analysis and qRT-PCR results showed that most *CmSUN*s are specifically enriched in melon reproductive organs, such as young flowers and ovaries. Through genetic transformation in melons, we found that overexpression of *CmSUN23-24* and *CmSUN25-26-27c* led to an increased fruit shape index, suggesting that they act as essential regulators in melon fruit shape variation. Subcellular localization revealed that the CmSUN23-24 protein is located in the cytoplasmic membrane. A direct interaction between CmSUN23-24 and a Calmodulin protein CmCaM5 was found by yeast two-hybrid assay, which indicated their participation in the calcium signal transduction pathway in regulating plant growth. These findings revealed the molecular characteristics, expression profile, and functional pattern of the *CmSUN* genes, and may provide the theoretical basis for the genetic improvement of melon fruit breeding.

## 1. Introduction

Melon (*Cucumis melo* L.) is a globally cultivated horticulture crop, bearing sweet and pleasant fruit with high nutritional value [1,2,3]. The long-term domestication process and wide distribution have produced multiple melon cultivars with diversified fruit traits, especially the fruit shape and size [4]. Fruit shape index (FSI) refers to the ratio of the fruit’s longitudinal diameter to the transverse diameter, and is an important agronomic trait that affects melon consumption [5]. Melon FSI can vary between round, oval, and long, and has always been used as a fruit quality screening standard to classify different market groups and satisfy consumer preferences [6]. In melon, the molecular mechanism underlying the outstanding diversity of FSI needs to be further studied [3]. The establishment of fruit shape is mediated by cell differentiation and cell expansion, which are caused by multiple endogenous gene activity [7]. Considerable research on the tomato has found that the *SUN* gene is one of the pivotal regulators in fruit shape regulation [8,9]. Overexpression of *SlSUN*/*SlIQD12*leads to long slender fruits with an increased number of vertical cells and a reduced number of horizontal cells, and *SlSUNs* regulate the expression of genes involved in cell division, cell wall development, and the auxin pathway [10,11].

*SUN* genes encode the IQD protein, a plant-specific calmodulin-binding protein that consists of 67 amino acid residues, containing three copies of the IQ motif and separated in precise spacing by short sequences of 11 and 15 amino acid residues. IQ67 domain usually contains 1~3 core ‘IQ’ motifs, 1~3 ‘1-8-14’ motifs, and 1~4 ‘1-5-10’ motifs [12,13]. The conserved motifs can bind to multifunctional calmodulin/calmodulin-like (CaM/CmL) proteins [14,15]. CaM/CmL transmits signals to downstream IQD receptor proteins through different forms of interaction with calcium signals [16,17,18,19,20,21,22]. In the *Arabidopsis thaliana*, the *SUN* family genes *AtIQD11*, *AtIQD14,* and *AtIQD16* have also been shown to regulate cell morphology by elongating or distorting cell shape [23].

*SUN* family genes participate in many aspects of plant development. *Arabidopsis AtIQD1* gene was the first to be discovered [12], and can activate the expression of glucosinolate metabolic pathway-related genes, thus increasing the glucosinolate content and improving the disease resistance [13]. Knockout of *AtIQD5* leads to variations in cell size, skeleton, and stability of microtubule [24]. *AtIQD18* is mediated by auxin signaling, and its overexpression results in a loose cell morphology phenotype [25]. The IQD role in microtubules may be mediated by a functional unknown domain, DUF4005 [26]. *SUN* genes have been involved in regulating cell shape and cell arrangement, which are closely related to the plant development and fruit shape index.

In recent years, the *SUN* family genes have been identified in many species. There are 33 *SUN* members in *Arabidopsis* [12], 29 members in *Oryza sativa* [12], 33 members in *Solanum lycopersicum* [27], 67 members in *Soybean* [28], 26 members in *Zea mays* [29], and 22 members in *Cucumis sativus* L. [30]. *CsSUN*, a homologous gene of tomato *SlSUN*/*SlIQD12*, was the candidate gene for the fruit shape of the cucumber QTL *FS1.2*, and its expression was lower in round-fruit cucumber than in long-fruit cucumber [31]. In watermelon, a 159 bp deletion in *ClSUN25-26-27a* underlies the difference between elongated fruit and spherical fruit [32,33]. Pan and Monforte mentioned 21 and 24 melon *SUN* members in the previous study, respectively [30,34]. In *Oryza sativa*, *OsIQD14* can control grain shape by regulating cell shape [35]. In other plants, such as soybean, maize, and Arabidopsis, IQD gene expression can be positively or negatively regulated by hormone treatments and abiotic stress [28,29,36].

In this study, we performed genome-wide identification and bioinformatics exploration of melon *SUN* family genes. The *CmSUN*s expression profile was analyzed by high-throughput sequencing data and verified by qRT-PCR. In the genome-wide association analysis of 297 melon germplasm, *CmSUN23-24* and *CmSUN25-26-27c* were presumed to be underlying the key loci of melon fruit shape [37]. Performing functional analyses through genetic transformation in melon, we found that overexpression of *CmSUN23-24* and *CmSUN25-26-27c* resulted in fruit shape variation. Furthermore, we identified a CaM protein that can directly interact with CmSUN23-24, suggesting that they contribute to fruit shape regulation through a regulatory gene network in melon.

## 2. Results

### 2.1. CmSUN Family Gene Identification and Sequence Analyses 

A total of 24 CmSUN members were identified by searching the Hidden Markov model PF00612 of the IQ motifs and blasting probe sequences in the melon protein database, including the 21 CmSUNs consistent with the previous study [30,34]. In the CmSUN family, CmSUN9-10a is the shortest protein consisting of 261 amino acids (aa), and the longest protein, CmSUN32b, is 845 aa in length. Physical and chemical analyses showed that the molecular weights of the corresponding proteins range from 29.9 to 93.4 k Dalton (kD), and the theoretical isoelectric points range from 5.59 (CmSUN32b) to 10.87 (CmSUN13-14a) (Table 1). The average theoretical isoelectric point of 24 CmSUN proteins was 9.94, indicating that they contain many basic amino acids.

**Figure 1:**
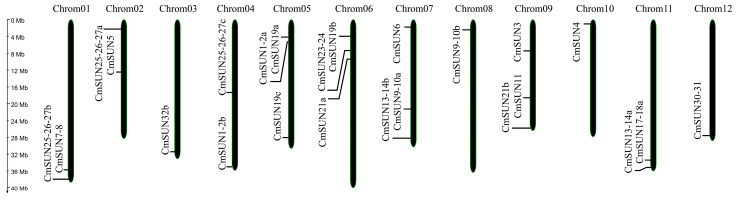
Distribution of 24 *CmSUN* genes on melon chromosomes.

**Figure 2:**
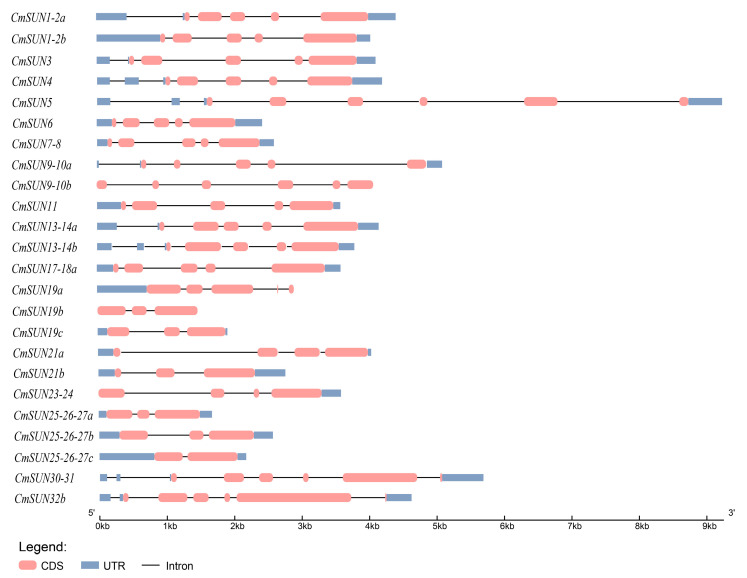
Gene structure of 24 *CmSUN* genes. The pink box represents the exon, the black lines represents the intron, blue box represents the UTR.

The 24 *CmSUN*s were unevenly distributed on 12 chromosomes (Figure 1). The gene structure analyses showed that all *CmSUN*s contained exons and introns. The number of exons in *CmSUN*s varies from 2 to 6. *CmSUN19b*, *19c*, *25-26-27a*, and *25-26-27b* have three exons that display similar-length arrangements in structures following a ‘medium—short—long’ pattern; *CmSUN1-2a*, *1-2b*, *3*, *4*, *6*, *7-8*, *11*, *13-14a*, *13-14b*, and *17-18a* have five exons that display similar-length arrangement in structures following a ‘short—medium—medium—short—long’ pattern; *CmSUN5*, *30-31*, and *32b* have six exons that display similar-length arrangements in structures following a ‘short—medium—medium—short—long—short’ pattern (Figure 2). The strong similarity of the three groups of *CmSUN* genes may indicate their conservation in the gene evolution process and function mode.

**Figure 3:**
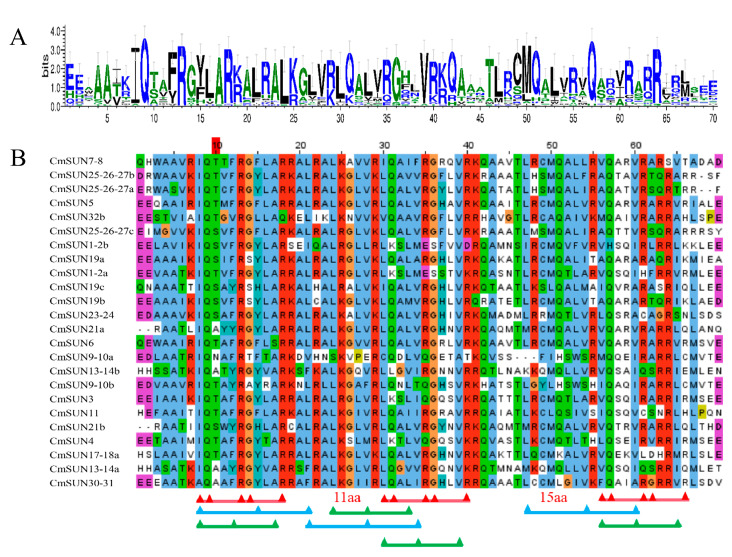
CmSUNs protein sequence analyses. (**A**) The logos show the conserved sites in IQ67 domain in CmSUN sequences. (**B**) Multiple sequence alignment of 24 CmSUN proteins. The red lines represent the ‘IQ’ core motif; The blue lines represent the ‘1-8-14’ motif; The green lines represent the ‘1-5-10’ motif; ‘11 aa’ and ‘15 aa’ represent the amino acid interval between the three ‘IQ’ motifs.

Through analyzing the CmSUNs protein domains, we found that arginine (R), isoleucine (I), glutamine (Q), and leucine (L) sites are highly conserved in different species (Figure 3A). Multiple sequence alignments of CmSUNs showed that all of the proteins contain a typical IQ67 conserved domain with 1~3 core ‘IQ’ motifs (IQXXXRGXXXR), or the less-restricted motifs ([ILV]QXXXRXXXR[RK]), 1~3 ‘1-8-14’ motifs ([FILVW]XXXXXX[FAILVW]XXXXX[FILVW]), and 1~4 ‘1-5-10’ motifs ([FILVW]XXX[FILV]XXXX[FILVW]). The three IQ motifs are precisely separated by 11 and 15 amino acids [12] (Figure 3B).

### 2.2. Phylogenetic Tree Analysis of SUN Family Proteins

**Figure 4:**
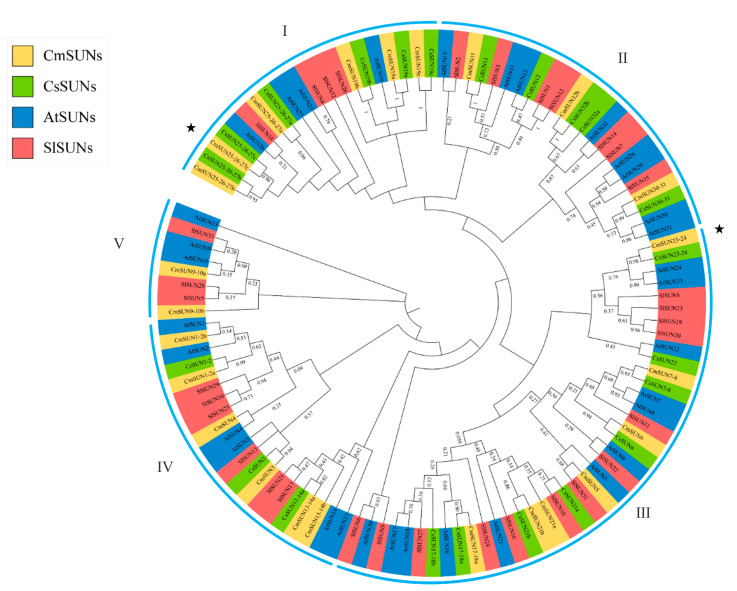
Phylogenetic tree analyses of SUN proteins from *Arabidopsis*, *Solanum Lycopersicon*, *Cucumis sativus*, and *Cucumis melo*. The blue represents AtSUNs, the green represents CsSUNs, the yellow represents CmSUNs, and the red represents SlSUNs. ‘I~V’ indicates different subfamily of SUN members in four species. The asterisk indicates the functionally studied CmSUN genes.

The phylogenetic tree was constructed by aligning the 33 AtSUNs in *Arabidopsis* [12], 22 CsSUNs in *Cucumis sativus* [30], 33 SlSUNs in *Solanum lycopersicum* [27], and 24 CmSUNs in *Cucumis melo*. The SUN family members can be divided into five clades I~V (Figure 4). CmSUN25-26-27a, 25-26-27b, and 25-26-27c are branched in the same clade with the fruit shape regulator gene CsSUN25-26-27a [31]. CmSUN23-24 and CmSUN25-26-27c are the homolog genes of CsSUN23-24 and CsSUN25-26-27c, which also participate in regulating fruit shape in cucumber [38]. CmSUN11 is homologous to the cell shape-related AtSUN11 gene; CmSUN1-2a and 1-2b are branched in the same clade with AtSUN1, which is the foundational SUN member in Arabidopsis.

### 2.3. Expression Profiling of CmSUN Genes

**Figure 5:**
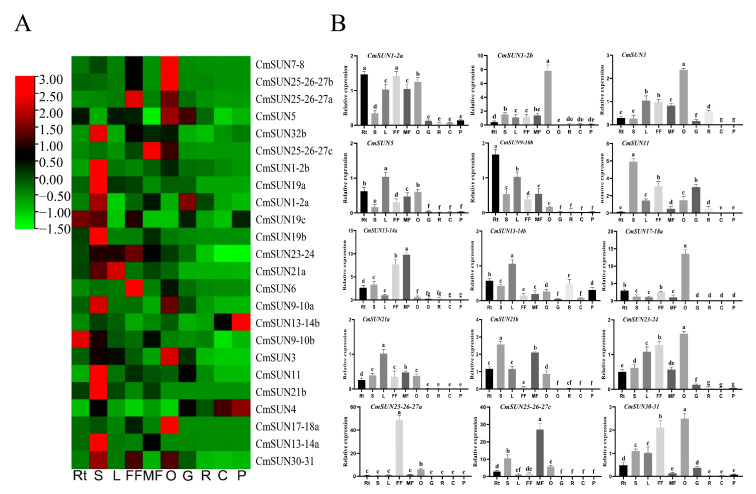
Expression analysis of *CmSUN*s in melon. (**A**) Heat map of 24 *CmSUN* gene expression in different melon tissues. (**B**) Expression profiles of part *CmSUN* genes in melon. Rt, S, L, FF, MF, O, G, R, C, and P represent root, stem, leaf, female flower, male flower, ovary, growing stage, ripening stage, climacteric stage, and post-climacteric stage, respectively. Three biological replicates and three technique replicates were performed for each qRT-PCR analysis. ‘a–g’ indicates the significance difference in the tissues.

The heat map of *CmSUN* expression was generated according to the transcriptomic analyses in different melon tissues (Figure 5A). The significant differences in relative expression in the ten different tissues are indicated by salient letter notation. The relatively high transcripts of the *CmSUN*s accumulated in melon stem, ovary, and flower. In the late development stages of the fruit, such as the growing stage (18 days after pollination, DAP), ripening stage (36 DAP), climacteric stage (determined according to breathalyzer), and post-climacteric stage (48 h after climacteric stage), the *CmSUN*s displayed low expression levels. We further verified the expression patterns of *CmSUN* genes by performing qRT-PCR in different melon tissues. The qRT-PCR results were consistent with the transcriptome analyses (Figure 5B). *CmSUN25-26-27a* has the highest expression in female flowers, and may have an active role in regulating gynoecium development; *CmSUN13-14a* and *CmSUN25-26-27c* are highly expressed in male flowers; *CmSUN1-2b*, *CmSUN3*, *CmSUN17-18a*, *CmSUN23-24*, and *CmSUN30-31* showed the highest expression in the ovary compared to the other tissues, suggesting their potential role in fruit development regulation.

### 2.4. Overexpression of CmSUN23-24 and CmSUN25-26-27c Resulted in Melon Fruit Shape Variation

**Figure 6:**
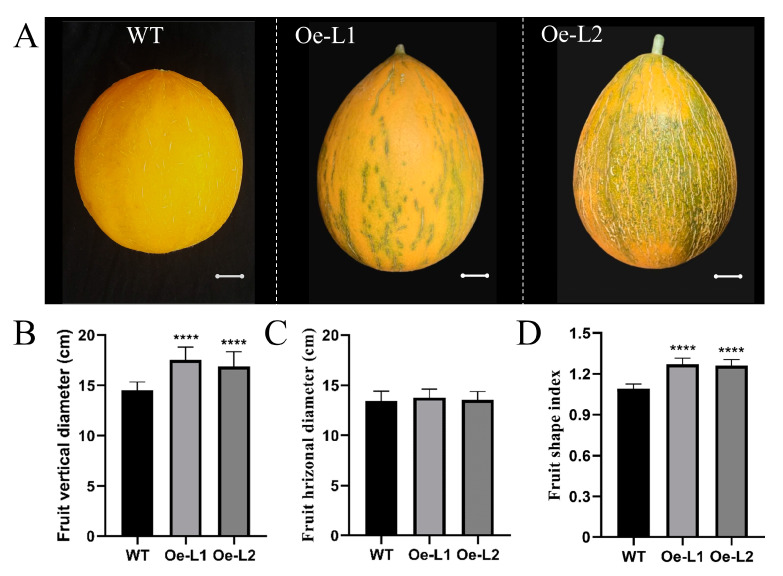
Phenotypic analysis of *CmSUN25-26-27c*-overexpressed transgenic lines. (**A**) Phenotypic observation of the fruits in transgenic lines. (**B**) Fruit vertical diameter significantly increased in transgenic lines. (**C**) Fruit horizonal diameter comparison between transgenic lines and WT. (**D**) Statistical analyses of fruit shape index in transgenic lines and WT. Scale bars = 2 cm. Oe-L1 and Oe-L2 indicate *CmSUN25-26-27c*-overexpressed transgenic lines. **** *p* value < 0.0001.

**Figure 7:**
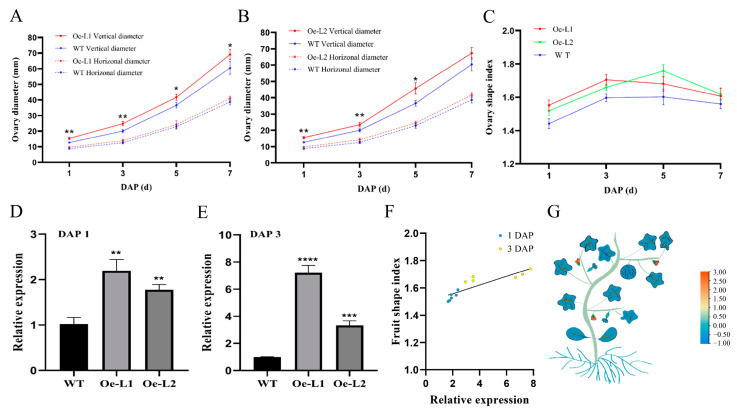
Statistical data of *CmSUN25-26-27c*-overexpressed transgenic fruits. (**A**,**B**) Vertical and horizonal diameter comparison of the fruits at 1 DAP (day after pollination), 3 DAP, 5 DAP, and 7 DAP in transgenic lines and WT. (**C**) Statistical analyses of ovary shape index in transgenic lines and WT in DAP 1~7. (**D**,**E**) qRT-PCR analyses of *CmSUN25-26-27c* expression in the ovary at 1 DAP and 3 DAP. Oe-L1 and Oe-L2 indicate *CmSUN25-26-27c*-overexpressed transgenic lines. (**F**) Scatterplot analysis shows the relevant of FSI and *CmSUN25-26-27c* expression. (**G**) Schematic model of relative expression of *CmSUN25-26-27c* in different melon tissues. * *p* value < 0.05; ** *p* value < 0.01; *** *p* value < 0.001; **** *p* value < 0.0001.

*CmSUN23-24* and *CmSUN25-26-27c* were presumed as candidate genes for melon fruit shape QTL [37]. To verify the role of *CmSUN23-24* and *CmSUN25-26-27c* in fruit regulation, we facilitated the transformation of the genes driven by the 35S promoter into the melon. We obtained six *CmSUN25-26-27c*-overexpressed transgenic lines, and two representative phenotypic lines were chosen for further characterization. Compared to the wild type plants, the vertical diameter of the *CmSUN25-26-27c-Oe* mature fruits was significantly increased, while the horizontal diameter showed no difference (Figure 6A–C). Thus, the melon fruit shape index is more enlarged in the transgenic lines than in WT (Figure 6D). In *CmSUN25-26-27c-Oe* lines, the fruit shape index was about 1.23 (Oe-L1, n > 20) and 1.21 (Oe-L2, n > 20), and the average FSI 1.22 was 11.9% larger than that in WT (1.09, n > 100). We measured the vertical and horizontal diameter of the transgenic fruits at DAP 1~7, which was the crucial time for determining the melon fruit shape. The data showed a similar fruit enlargement curve in the WT and transgenic lines (Figure 7A–C). *CmSUN25-26-27c-Oe* lines had a higher FSI at early fruit elongation stages than WT, particularly in the fruits at 1 DAP and 3 DAP (Figure 7A–C). Then we examined *CmSUN25-26-27c* expression in the ovaries at 1 DAP and found twofold elevated expression in the overexpression lines (Figure 7D). In the ovaries at 3 DAP, *CmSUN25-26-27c* expression was increased by about 7.2 and 3.3 times in Oe-L1 and Oe-L2 lines compared to the WT, respectively (Figure 7E). A scatterplot analysis was performed to verify the relevant between FSI and *CmSUN25-26-27c* expression (Figure 7F). The correlation coefficient indicates the significant relevance (R^2^ = 0.76) between gene expression and FSI (Figure 7F). In the transcriptomic analyses, *CmSUN25-26-27c* transcripts were also highly accumulated in the male flower (Figure 7G). The phenotypic analyses suggested that the *CmSUN25-26-27c* gene positively regulated melon fruit elongation and functions in the early developmental phase.

**Figure 8:**
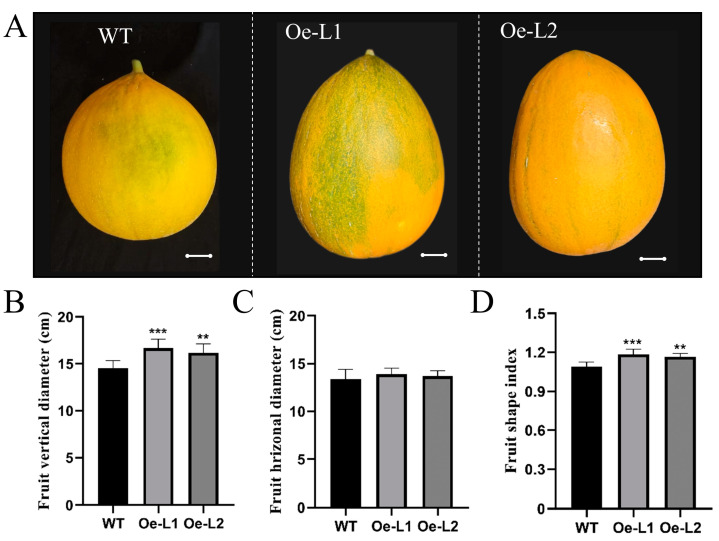
Phenotypic analysis of *CmSUN23-24*-overexpressed transgenic lines. (**A**) Phenotypic observation of the fruits in transgenic lines. (**B**) Fruit vertical diameter significantly increased in transgenic lines. (**C**) Fruit horizonal diameter comparison between transgenic lines and WT. (**D**) Statistical analyses of fruit shape index in transgenic lines and WT. Scale bars = 2 cm. Oe-L1 and Oe-L2 indicate *CmSUN23-24*-overexpressed transgenic lines. ** *p* value < 0.01; *** *p* value < 0.001.

**Figure 9:**
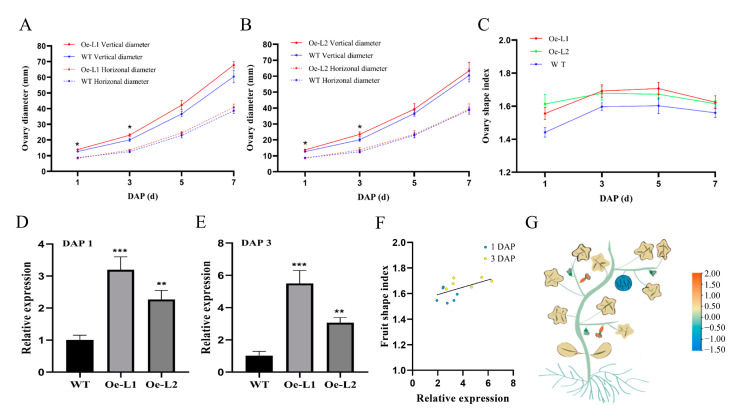
Statistical data of *CmSUN23-24*-overexpressed in transgenic fruits. (**A**,**B**) Vertical and horizonal diameter comparison of the fruits at 1 DAP, 3 DAP, 5 DAP, and 7 DAP in transgenic lines and WT. (**C**) Statistical analyses of ovary shape index in transgenic lines and WT in DAP 1~7. (**D**,**E**) qRT-PCR analyses of *CmSUN23-24* expression in the ovary at 1 DAP and 3 DAP. Oe-L1 and Oe-L2 indicate *CmSUN23-24*-overexpressed transgenic lines. (**F**) Scatterplot analysis showed the positive relevant of FSI and *CmSUN23-24* expression. (**G**) Schematic model of relative expression of *CmSUN23-24* in different melon tissues. * *p* value < 0.05; ** *p* value < 0.01; *** *p* value < 0.001.

In *CmSUN23-24-Oe* lines, the altered vertical diameter and unchanged horizontal diameter resulted in the increased fruit shape index (Figure 8A–D). The average FSI (1.18, n > 40) of the mature fruits in *CmSUN23-24-Oe* lines was 8.3% larger than in WT (1.09, n > 100) (Figure 8D). *CmSUN23-24-Oe* lines also had increased vertical diameter and FSI at early developmental stages (Figure 9A–C). *CmSUN23-24* expression was dramatically increased at 1 DAP and 3 DAP in ovaries in the severe line (Figure 9D,E). The *CmSUN23-24* expression showed a weak relevance (R^2^ = 0.31) with the FSI (Figure 9F). The transcriptomic analyses in different melon tissues showed that *CmSUN23-24* was highly accumulated in female flowers and ovary (Figure 9G). The phenotype of *CmSUN23-24-Oe* lines is similar to *CmSUN25-26-27c-Oe* lines, indicating their redundant function in mediating melon fruit size development.

### 2.5. Yeast Two-Hybrid and Subcellular Localization

**Figure 10:**
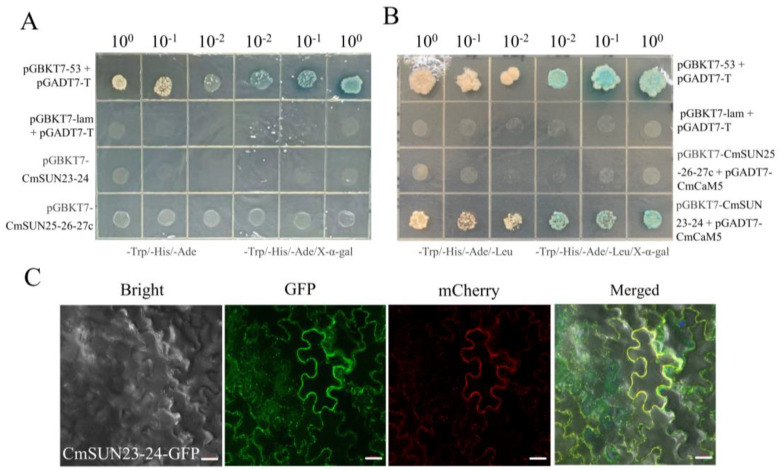
CmSUN23-24 has a protein interaction with CmCaM5. (**A**) Transcriptional activation of CmSUN23-24 and CmSUN25-26-27c protein. pGBKT7-53 + pGADT7-T and pGBKT7-lam + pGADT7-T were served as positive and negative control, respectively. The values 10^0^, 10^1^ and 10^2^ indicate different concentration solutions; -Trp/-His/-Ade indicates DO supplement; X-α-gal was a chromogenic substrate of yeast galactosidase. (**B**) Yeast-two hybrid assays were performed between CmSUN23-24, CmSUN25-26-27c, and CmCaM5. (**C**) Subcellular localization of CmSUN23-24 in tobacco leaves. Bright indicates light field; GFP indicates green fluorescence excitation field; mCherry indicates fluorescence excitation field; Merged indicates the overlapping of the three fields. Scale bar = 20 μm.

The transcriptional activation activity of CmSUN23-24 and CmSUN25-26-27c proteins were examined. We found that CmSUN23-24 and CmSUN25-26-27c did not contain transcriptional activation activity (Figure 10A). CmCaM5 and CmCmL11 are homologous proteins of AtCaM1, which can interact with several IQD proteins. The Y2H result showed that CmSUN23-24 had a direct interaction with CmCaM5 protein (Figure 10B). However, there was no interaction between CmSUN25-26-27c, CmCaM5 and CmCmL11. We performed subcellular localization in tobacco leaves and found that CmSUN23-24 was localized in the cell membrane (Figure 10C).

## 3. Discussion

*SUN* family genes participate in plant disease defense [13], stress resistance, plant development [28,29,36], cell arrangement, and cytoskeleton regulation [8,9,24,25]. *Arabidopsis* [12] and *Solanum lycopersicum* [27] have nearly 30 members, and *Soybean* has 67 more members [28]. In previous studies, Monforte identified 24 CmSUNs in melon by blasting the protein sequences of the known tomato SUN genes into the melon genome [34]; Pan mentioned 21 proteins containing both IQ67 and DUF4005 domains and identified CmSUNs by their homology with the *Arabidopsis* genes [30]. The integrity of the IQ67 domain is crucial in binding with the Ca^2+^ receptors Calmodulin/Calmodulin-like (CaM/CmL) [39]. However, some SUN members were identified incompletely due to the low conservation of the IQ67 domain in sequence and quantity during evolution. In this study, we considered the protein containing an integrated and typical conserved IQ67 domain with three IQ motifs precisely separated by 11 and 15 amino acid residues to be the *CmSUN* genes, following the same criteria as the *SUN* family gene identification in *Arabidopsis* and *Oryza sativa* [12]. We characterized 24 *CmSUN* members, including 21 consensus genes with the previous work. Moreover, we found two other protein families, Myosin (7 members) and CaMTA (2 members), and several proteins that contained IQ motifs. It is noteworthy that nine genes of the Myosin and CaMTA family contain 1–3 IQ motifs, but possess unique amino acid spacing patterns. The two gene families have long amino acid sequences (744–1523 aa), high molecular weights (84.7–172.8 kD), and average low isoelectric points (7.6). CmSUNs are relatively short in length, have a high average isoelectric point (9.9), and contain a typical conserved IQ67 domain and a conserved exon-intron structure. During the evolution, the first IQ motif is the most conservative in the sequences, and the second and third IQ are more distinctive [12]. In our preliminary results, MELO3C005949.2.1 and MELO3C005636.2.1, which contain two IQ motifs and the specific intervals, were also annotated as IQD proteins. However, considering the absence of the first IQ motif and low sequence conservation, we did not nominate the two genes as *CmSUN* members. Taken together, our solid work on identifying *CmSUN* family members in melon provided a theoretical foundation for further characterization of their function and molecular mechanism.

Expression analyses in melon tissues showed that *CmSUN* transcripts were mainly accumulated in the lateral organs and the young fruits. Most members tended to be highly expressed in flower and ovary, and gradually disappeared in the fruit at the late developmental stage. In cucumber, *CsSUN30-31* had a high expression level in various tissues, which may have been involved in the many aspects of plant growth. The expression of *CsSUN*, *CsSUN17-18b*, and other three *SUN* genes was correlated with cucumber fruit development. *CsSUN23-24* and *CsSUN25-26-27c* expression were significantly higher in long fruit than short fruit, while *CsSUN21a* and the other two genes had much higher expression in short fruit [38], meaning they had the opposite role in regulating fruit elongation. In tomato, *SlSUN1* and *SlSUN28* showed high expression in growing fruits; *SlSUN5*, *SlSUN11*, *SlSUN12*, *SlSUN21*, *SlSUN22*, and *SlSUN27* were expressed actively in vegetative growth [27]. The expression pattern of *SlSUN*s in tomato was similar to *CmSUN*s in different melon tissues, which may imply their conservative function in horticulture plant development. 

In the functional analyses, we revealed that the overexpression of *CmSUN23-24* and *CmSUN25-26-27c* resulted in elongated fruits and increased fruit shape index, suggesting that they had an important role in melon fruit shape regulation. In cucumber, *CsSUN* (*CsSUN25-26-27a*) is a positive regulator in fruit elongation, and the phenotype has a consistency with its expression in fruits of different lengths [38]. In watermelon, *ClSUN25-26-27a* is a pivotal factor in regulating the round or elongated fruit shape [32,33]. In melon, *CmSUN25-26-27c* is homologous to *CsSUN25-26-27a*, *CsSUN25-26-27c*, and *ClSUN25-26-27a*. Overexpression of *CmSUN23-24* and *CmSUN25-26-27c* altered the fruit shape at the early ovary developmental stage. The increased expression level, enlarged fruit shape index, and significant correlation coefficient in melon transgenic fruits demonstrated that *CmSUN23-24* and *CmSUN25-26-27c* mediate fruit shape index variation. 

*SUN* family genes usually act as downstream targets in the calcium signal transduction pathway, which contributes to various physiological activities in plant development [40,41,42,43]. Most *CmSUN*s are rich in basic amino acids and hydrophobic amino acids and provide advantages for them to bind with the Ca^2+^ receptors Calmodulin/Calmodulin-like (CaM/CmL) [23,35]. Through performing the yeast two-hybrid assay, we found that CmSUN23-24 can directly bind to CmCaM5. CmSUN23-24 localized in the cell membrane, which may be regulated by the CaM and Ca^2+^ binding complex in Ca^2+^ signal transduction, and then participated in melon plant development. In *Arabidopsis*, IQD1 localized in microtubules and interacted with KLCR1 (kinesin light chain-related) protein or CaM2 to recruit them for cell division regulation [39]. AtIQD5 and AtIQD18 can also regulate the cell size and cell shape by protein interaction with microtubulin and then affecting the microtubule dynamics [24,25]. The combination between IQD and microtubulin was mediated by a DUF4005 domain [26]. CmSUN23-24 and CmSUN25-26-27c both contained the DUF4005 domain. Therefore, we speculated that CmSUN23-24 positively regulates melon fruit elongation through the CmCaM5-dependent Ca^2+^ signaling transduction pathway and may have a positive regulation in microtubules. Although CmSUN25-26-27c has the potential CaM binding sites, there was no interaction between CmSUN25-26-27c and CmCaM5, implying that there may other CmCaMs/CmLs working with CmSUN25-26-27c and then participating in the calcium signal and microtubule regulation.

## 4. Materials and Methods

### 4.1. Identification of SUN Gene Family Members

The melon protein sequence file (CM3.6.1_pep) was downloaded from the CuGenDB (http://cucurbitgenomics.org/, accessed on 5 January 2022) online website [44], which refers to the melon genome reported by Garcia-Mas [45]. The Hidden Markov model file (PF00612) of the IQ motif was downloaded from the Pfam (http://pfam.xfam.org/, accessed on 5 January 2022) database [46]. The IQD protein sequences of *Arabidopsis*, *Solanum lycopersicum* and *Cucumis sativus* was downloaded from previous study [12]. We searched the melon IQD proteins in HMM (http://www.hmmer.org/, accessed on 6 January 2022) software [47] using the Hidden Markov model (PF00612) and blasted the melon protein data using IQD sequences from Arabidopsis, tomato and cucumber as the probe in CuGenDB. Redundant sequences were removed and the remaining sequences were aligned through the online websites of Pfam (http://pfam.xfam.org/, accessed on 9 January 2022) and Smart (http://smart.embl-heidelberg.de/, accessed on 9 January 2022) [48] to verify the existence of the IQ motifs. The integrity of IQ67 conserved domain was demonstrated by multi-sequence alignment in MEGA7 software [49]. The protein sequences with three IQ motifs separated by 11 and 15 amino acid residues were identified as CmSUNs.

### 4.2. Sequence Analysis and Phylogenetic Tree Construction 

Open reading frame (ORF) length, molecular weight (MW) and theoretical isoelectric point (pI) of *CmSUN* members were analyzed by ExPASy online website (http://web.expasy.org/protparam/, accessed on 12 January 2022) [50]. Location information of *CmSUN* members on chromosome was obtained from CuGenDB, and the chromosome map was drawn by the MG2C (http://mg2c.iask.in/mg2c_v2.0/, accessed on 16 January 2022) [51]. The CmSUN protein sequences were aligned by the Clustal W program in MEGA7 and visualized by Jalview [52]. Conserved sites of CmSUN protein domain were predicted by online software WEBLOGO (http://weblogo.berkeley.edu/, accessed on 18 January 2022) [53]. The *CmSUN* gene structure information (intron-exon) was obtained from the GFF file (CM3.6.1_gene.gff3) in the CuGenDB, and visualized by GSDS (http://gsds.cbi.pku.edu.cn/, accessed on 20 January 2022) [54]. The neighbor-joining method in MEGA7 software was utilized to construct a phylogenetic tree. The bootstrap value was 1000. SUN members in *Arabidopsis*, *Cucumis sativus*, *Solanum lycopersicum*, and *Cucumis melo* were compared. The phylogenetic tree was visualized through iTOL online software (https://itol.embl.de/, accessed on 28 January 2022) [55].

### 4.3. Plant Materials and Growth Conditions

The melon (*Cucumis melo* cv. Hetao) inbred lines were cultivated in Dengkou county of Inner Mongolia region and used in this study. All plant materials were provided by Hasi lab at Inner Mongolia University, Hohhot, China. The melon seedlings grew in an artificial climate chamber under the conditions of 60% humidity, 16 h light (25 °C), and 8 h dark (18 °C). The female fruits at anthesis day were self-pollinated. The female flower, male flower, ovary, fruits from four different developmental stages, roots, stems, and leaves were collected for expression analyses. Samples from the young lateral root, the second or third section of stem, the 3 to 5 true leaves, the unpollinated female flower, and male flowers at anthesis, and the fruit mesocarp were frozen in liquid nitrogen and stored at −80 °C. The tissues were sampled from different plants at same growth phase.

### 4.4. Expression Analysis and Quantitative Real-Time PCR 

Different tissues from melon were used for the total RNA extraction. Raw reads for RNA-Seq data were quoted from our previous work [56]. The expression level of *CmSUN*s in the above ten periods was obtained from statistical data analyses. The heat map was drawn by using TBtools software [57], and parameters were set as default. 

The relative expression level of *CmSUN23-24* and *CmSUN25-26-27c* in the transgenic melon were verified by qRT-PCR. Primers were designed by Primer 5. The primer information was listed in Supplemental Table S1. RNA was reversed to cDNA by using the PrimeScript™ RT Reagent Kit and gDNA Eraser Kit (Takara Bio, Shiga, Japan). SYBR^®^ Premix Ex Taq™ II (Takara Bio, Shiga, Japan) and 96-well Chromo4 Real-Time PCR System were used for qRT-PCR and 2^−∆∆CT^ method was utilized for expression level analyses. Three biological replicates and three technical replicates were performed for each gene.

### 4.5. Gene Cloning and Plant Transformation

The whole-length coding sequence of *CmSUN23-24* (MELO3C006884) and *CmSUN25-26-27c* (MELO3C013004) were amplified by gene-specific primers. The primers are listed in Supplemental Table S1. The restriction sites *Xba*I and *Bam*HI were used for constructing the overexpression vector. The target gene was recombined into the overexpression vector pCAMBIA-1305 driven by 35S promoter. To obtain transgenic plants, the recombinant plasmids were diluted to 100 ng/μL with 2 × SSC solution (sodium citrate buffer, pH = 7.0) and ddH_2_O. After 7 h in artificial pollination, 10 μL plasmid solution was injected into the melon fruits as described previously [58]. Fruits of the T_1_ generation transgenic plants were sampled and detected by PCR with a pair of specific primers designed on pCAMBIA-1305 vector.

### 4.6. Subcellular Localization 

The full-length coding sequence of *CmSUN23-24* without the termination codon was jointed to the vector pCAMBIA-1300 at the upstream of GFP and transformed into agrobacterium GV3101. The primers are listed in Supplemental Table S1. The lower epidermis of tobacco leaves was infected for subcellular localization experiments. A confocal laser-scanning microscope was used for observing the fluorescence signals under 488-nm wave length excitation. 

### 4.7. Yeast Two-Hybrid Assay 

Full lengths of *CmSUN23-24* and *CmSUN25-26-27c* were constructed to pGBKT7 vectors. Full lengths of *CmCaM5* (MELO3C014698) and *CmCmL11* (MELO3C006491) were constructed to pGBKT7 and pGADT7 vectors, respectively. The pGBKT7 recombinant plasmids were transformed into the yeast strain AH109 (TaKaRa). SD/-Trp/-His/-Ade solid medium with 4 mg/mL X-α-gal and without X-α-gal was used for the activation test. Concentrations of 10^0^, 10^−1^, and 10^−2^ of bacterial concentrations were used for selecting interacted combination. pGBKT7-53+pGADT7-T served as positive control; pGBKT7-lam+pGADT7-T was used as negative control. CmSUN23-24-pGBKT7 or CmSUN25-26-27c-pGBKT7 were co-transformed with CmCaM5-pGADT7 or CmCmL11-pGADT7.

## 5. Conclusions

We identified 24 *SUN* family genes in melon, and performed a comprehensive analysis. Twenty-four CmSUN members all contained typical IQ67 motifs precisely separated by 11 and 15 amino acids. Most *CmSUNs* transcripts were accumulated in the melon stem, ovary, and flower. The biological function of *CmSUN23-24* and *CmSUN25-26-27c* in regulating melon fruit shape was revealed by the gene transformation analysis. Protein interaction between CmSUN23-24 and CmCaM5 suggested their participation in the Ca^2+^ signaling transduction pathway. Future studies on the function and molecular mechanism of *CmSUN*s would be valuable for uncovering the gene regulation in fruit development in melon.

## Figures and Tables

**Table 1:** Information and physicochemical properties of 24 *CmSUN* members.

Name	Gene ID	Chromosome Distribution	ORF (bp)	Amino Acid Length (aa)	MW (kD)	pI
*CmSUN1-2a*	MELO3C014258.2.1	Chrom05	1473	490	53.7	9.87
*CmSUN1-2b*	MELO3C009321.2.1	Chrom04	1491	496	56.4	10.34
*CmSUN3*	MELO3C025505.2.1	Chrom09	1449	482	53.4	10.42
*CmSUN4*	MELO3C012442.2.1	Chrom10	1395	464	52.1	10.06
*CmSUN5*	MELO3C009991.2.1	Chrom02	1302	433	48.5	10.22
*CmSUN6*	MELO3C016880.2.1	Chrom07	1338	445	49.6	10.47
*CmSUN7-8*	MELO3C024381.2.1	Chrom01	1221	406	45.8	10.26
*CmSUN9-10a*	MELO3C016091.2.1	Chrom07	786	261	29.9	10.04
*CmSUN9-10b*	MELO3C007235.2.1	Chrom08	1107	368	41.9	10.31
*CmSUN11*	MELO3C005137.2.1	Chrom09	1440	479	54.1	10.02
*CmSUN13-14a*	MELO3C022423.2.1	Chrom11	1614	537	60.5	10.87
*CmSUN13-14b*	MELO3C017768.2.1	Chrom07	1650	549	61.4	10.47
*CmSUN17-18a*	MELO3C022253.2.1	Chrom11	1539	512	57.5	10.42
*CmSUN19a*	MELO3C014290.2.1	Chrom05	1452	483	53.8	9.63
*CmSUN19b*	MELO3C006504.2.1	Chrom06	1269	422	46.9	9.81
*CmSUN19c*	MELO3C004368.2.1	Chrom05	1128	375	43.4	9.98
*CmSUN21a*	MELO3C008499.2.1	Chrom06	1410	469	52.8	9.60
*CmSUN21b*	MELO3C005888.2.1	Chrom09	1125	374	41.8	9.96
*CmSUN23-24*	MELO3C006884.2.1	Chrom06	1413	470	51.5	10.28
*CmSUN25-26-27a*	MELO3C015418.2.1	Chrom02	1233	410	45.4	10.25
*CmSUN25-26-27b*	MELO3C024434.2.1	Chrom01	1293	430	48.3	9.66
*CmSUN25-26-27c*	MELO3C013004.2.1	Chrom04	1161	386	42.8	10.29
*CmSUN30-31*	MELO3C002201.2.1	Chrom12	1800	599	65.9	9.80
*CmSUN32b*	MELO3C010997.2.1	Chrom03	2538	845	93.4	5.59

## Data Availability

All the data and plant materials in relation to this work can be obtained through contacting with the corresponding author Gen Che (chegen@imu.edu.cn).

## Supplementary Materials

The supporting information can be downloaded at: https://www.mdpi.com/article/10.3390/ijms232416047/s1.
