# Supplementary material for: Genome-Wide Identification of the SUN Gene Family in Melon (Cucumis melo) and Functional Characterization of Two CmSUN Genes in Regulating Fruit Shape Variation
Source: Int J Mol Sci. 2022 Dec 16;23(24):16047. doi: 10.3390/ijms232416047 (PMC9785357; doi:10.3390/ijms232416047)
Supplement: Supplementary file 1 [file ijms-23-16047-s001.zip › ijms-2045131-supplementary.pdf]

Table S1 Primer sequence associated with this article experiments.

**Primer of gene clone**		
CmSUN23-24	F: GCTCTAGAATGGGCTTTCTCCGCC	R:CGGGATCCGATCATCTTGATTTGGTACTTGCA
CmSUN25-26-27c	F:GCTCTAGAATGGGGAAAGCTACAAGGT	R:CGGGATCCTCAAAATTCATCAAACCCT
**Primer of subcellular localization**		
CmSUN23-24	F: GGATCCATGGGCTTTCTCCGC	R:GCTCTAGATCTTGATTTGGTACTTGC
CmSUN25-26-27c	F:GGGGTACCATGGGGAAAGCTACAAGG	R:CGGGATCCAAATTCATCAAACCCTTC
**Primer of yeast two-hybrid**		
pGBKT7-CmSUN23-24	F:CGGGATCCATGGGCTTTCTCCGCC	R:AACTGCAGGATCATCTTGATTTGGTACTTGCA
pGBKT7-CmSUN25-26-27c	F:GGAATTC CATATGATGGGGAAAGCTACAAGGT	R:CGGGATCCTCAAAATTCATCAAACCCT
pGBKT7-CmCAM5	F:CGGGATCCCCTTCCTCATCAATTTCCATGG	R:AACTGCAGCAGTTCGATTGATTCTCACTTGGC
pGBKT7-CmCML11	F:CGGGATCCCTGAAGAAAAAAAACATGACGGAAG	R:AACTGCAGAAGCTACTTTTGAAACAATCATCCA
pGADT7-CmCAM5	F:GGAATTCCCTTCCTCATCAATTTCCATGG	R:CGGGATCCCAGTTCGATTGATTCTCACTTGGC
pGADT7-CmCML11	F:GGAATTCCATATGCTGAAGAAAAAAAACATGACGGAAG	R:CGGGATCCAAGCTACTTTTGAAACAATCATCCA
**The qRT-PCR primer of *CmSUNs* and reference gene**		
CmSUN1-2a	F:GAGTTCTACTGTGAAACGCCAAG	R:TCCTCCAACATCCTAACCCTC
CmSUN1-2b	F:CCCACTCCTTTTGTGACTATTGC	R:TTGGTGGCAGCGGTAGTAGAC

CmSUN3	F:TCGTCTATGGGAAACTAAGAGCAC	R:TTGGAAACGGCTTGATGATGT
CmSUN5	F:ACTGCTGCCTCGCATTCTG	R:CTCAGGTGCCAGTTTGTGCTT
CmSUN9-10b	F:ATTCAGACAGCATACCGAGCAT	R:CTTTCTAACGGAATGACCTTGAGT
CmSUN11	F:CCACCAAAAGCAACGACGAC	R:CGACCTCAACAGCAGCCTTAG
CmSUN13-14a	F:CATTCCTCTATTTAGGGAGCCAAG	R:GAGGTGTTGGTGGTGAAGAAGG
CmSUN13-14b	F:AGAGATTTTTCACTTGTCAGTCAGG	R:GACACTGCTCGGCTTCCTAA
CmSUN17-18a	F:CCAATGGATGGCAAAGAAGC	R:GGGAGATAAGTAGGAGTAGGGTCG
CmSUN21a	F:CGACGCCAAGGATTGAAGG	R:CATAGCCAGCCAAGCGAACT
CmSUN21b	F:GGAGGAGGCAGTTGGTTCTT	R:CATTTCTTAGCAGATTGAGGAGG
CmSUN23-24	F:ATTTGACAACTCGGCACTTCTG	R:TCTAACCAACTTGACCCGACTG
CmSUN25-26-27a	F:TTTCTTCTCCACTCCCTTGTCG	R:ATCGTGGAGTGCTCTGTGCC
CmSUN25-26-27c	F:GAGAAGGGTATTATCGTGGGTATG	R:CCATTATCTCATTCAACGCCACT
CmSUN30-31	F:GCCAAGGCATCGGAAACTAC	R:GGTAACTTTTGGCACCCCTTC
CmGAPDH	F:GTTTTCACCGACAAAGACAAAGCTG	R:TCCGGCTTGTATTCCTTCTCGTTAAC
